# Diversity as an individual resource in care work of the future: a scoping review protocol

**DOI:** 10.3389/fpubh.2026.1892688

**Published:** 2026-10-20

**Authors:** Mariya Lorke, Vivian Carstensen

**Affiliations:** 1 University of Applied Sciences and Arts (HSBI), Bielefeld, Germany; 2 Leibniz University Hannover, Hanover, Germany

**Keywords:** care workers, diverse, healthcare of the future, human capital theory, person-centred care

## Abstract

**Background:**

Evidence suggests that appropriately managed diversity in healthcare teams can enhance problem-solving, resilience, job satisfaction – all crucial for workforce retention. This scoping review aims to map evidence on how lived diversity experience is conceptualized and operationalized as an individual resource among care workers.

**Methods:**

An interdisciplinary approach across social anthropology, health sciences, and labor economics will be adopted. The databases to be searched include MEDLINE/PubMed, PsycINFO, Scopus and Web of Science and sources of grey literature. A two-stage screening process will be followed by systematic data extraction, with findings synthesized through descriptive numerical summaries and qualitative thematic analysis.

**Results:**

This scoping review is expected to shift the focus from organisational-level diversity research to research on how diversity contributes as an individual resource in care work contexts. It will map how diversity-as-resource is theoretically operationalized across disciplines, identifying existing measurement approaches and potentially revealing critical gaps in current assessment methods.

**Conclusion:**

By synthesizing evidence on how care workers’ lived diversity experiences are deployed as valuable competencies in care delivery, this review will provide foundational knowledge to inform workforce transformational strategies.

## Background

According to the estimations of the World Health Organisation (1), by 2030 the global shortage of healthcare professionals will amount to 10 million whilst, in many countries, the aging of the population perpetuates rising demand for health services, particularly in geriatric and long-term care. With an aging care workforce, increasing internal and external migration and growing deterioration of care workers’ health (1), the future of care work faces challenges of both scale and skill that impact needs, specialization, and lifelong learning requirements of health care professionals. These challenges are accompanied by structural issues such as high labor turnover, burnout, and the generally low esteem of care work that complicate recruitment, further training and retention of staff, thereby impeding quality assurance of care despite the crucial societal need (2). In this article, care refers to both health care (services addressing physical and mental health needs) and health-related social care (services supporting individual’s social, practical and welfare needs), reflecting the oftenoverlapping nature of these domains in practice.

Addressing the care crisis, requires not only well-structured action, including improving working conditions (by considering mental health and gender equality issues) and optimizing performance (by implementing digital technologies, redefine teams and support skill development) (1); it also demands a closer look at the changing composition of the workforce itself. Generation Z, e.g., born between 1997 and 2012, presents a distinct professional typus: a strong orientation toward work-life balance, active leisure, mental health, and opportunities for personal development (3–5), alongside digital socialization that leads to openness toward digitalization in nursing, whereby digital literacy is seen as a necessary skill requirement for modern nursing work (6). Crucially, this generation also determines a workforce that is increasingly diverse (across culture, background, and lived experiences) and evidence suggests that Gen Z places particular value on workplace diversity and inclusion (7). Inclusive leadership that actively embraces this diversity has been shown to positively impact work performance and motivation (8). In other words, the changing workforce does not only represent a challenge in numbers and skills, it also carries resources that, if acknowledged and cultivated, may offer new ways of responding to the crisis.

The major drivers of change shaping the care work of the future – demography, technology and economic trends – risk deepening the crisis we are already witnessing today. Tackling this requires both systemic and individual approaches. In particular, there is a need of resource-oriented (9) and people-centered frameworks (10) in health care that move beyond deficit-oriented thinking. This scoping review explores one such possibility – namely deploying individual lived diversity experiences as a resource in health care work of the future.

Accordingly, health care teams are typically multiprofessional and diverse in several dimensions (11) and there is a shift from fragmented, disease-centered care to integrated, person-centered care models (12). Therefore, it is valuable to examine if diversity can be deployed as an individual resource to improve both mental health and inclusion and increase team performance in the long run. Furthermore, care work can be expected to become more digitalized and technologized, with care workers navigating emerging technologies like robotic devices and AI while operating across non-traditional care settings. Such challenges add a new dimension to care workers’ already complex profiles – digital literacy (13) and positive attitudes toward technology. With increasing numbers of students from divergent backgrounds entering health studies, it is essential to consider the epistemic complexity resulting from the multifaceted perspectives that characterize the organization of health care.

Against this background, operationalizing diversity in the context of the care crisis seems essential. On the one hand, diversity as an individual resource represents exactly the kind of human-centric, adaptive capability and flexibility that defines valuable work, creating cultural intelligence that AI cannot replicate (and replace). On the other hand, it fuels the capabilities of creative problem-solving (14) and critical thinking that care work of the future requires. Diversity as an individual resource can also positively impact resilience (15), well-being and job satisfaction. In younger generations, valuing diversity may foster key drivers of motivation: recognition, appreciation, well-being, and skills (16). These aspects are crucial for voluntarily maintaining one’s occupation of paid care work. Existing literature indicates that if managed properly cultural diversity in health care teams can build an asset to healthcare organizations (11). Yet despite the body of evidence, the literature remains fragmented and lacks a clear understanding of the circumstances under which diversity de facto determines an asset among care workers. Given the breadth of disciplines in which diversity is theorized and discussed, this review focuses on literature situated in its disciplinary scope, within social anthropology, health sciences and labor economics.

The objective of this scoping review is to assess the extent of the literature and map evidence on how lived diversity experience is conceptualized and operationalized as an individual resource among health care workers. In this review, diversity is understood as lived experiences shaped by individual’s identity, emotions, social context, class, race, gender and other intersecting factors, rather than as knowledge acquired solely through diversity training. This lived, intersectional perspective is essential, as it moves beyond examining single diversity characteristics in isolation to consider their interconnectedness. Diversity characteristics are considered not individually, but in terms of how they interact and interrelate within the individual (17). This review aims at synthesizing both available evidence and conceptual approaches deployed to frame diversity as an individual resource. It should add to human capital research, address urgent workforce questions (including labor and future skills scarcity) and fill a gap at intersection of literatures. We need to (1) identify what types of diversity experiences are discussed as individual resources in care work, (2) examine conceptual frameworks for diversity as an individual resource and developing a theoretical model based on that, (3) explore how diversity-as-resource is measured or assessed and (4) identify outcomes associated with diversity as an individual resource.

The theoretical fundament of this scoping review is multifold. It builds on (i) diversity as a resource, (ii) an enhanced human capital framework that embeds diversity as (a dimension of) noncognitive endowment (18), and (iii) healthcare work of the future models. In the following, we explain how we define the key terms of this scoping review and analyze the interdisciplinary connections between them.

### Diversity as a resource

Considering critiques of the limitations of diversity, equity and inclusion (DEI) work in practice and literature (19), we argue it is essential to operationalize diversity as an individual resource when conducting this scoping review. We ground this approach in mainly three strands of reasoning. First, DEI measures and practices still favor traditional approaches aimed at correcting injustices from the past. These approaches remain primarily oriented toward the antidiscrimination policies established in the 1980s. In Germany, for example, institutions and companies sign the Charter of Diversity (20) committing themselves to fostering acknowledging and prejudice-free working environments. Hereby, diversity is seen as factor that is suspected to disrupt system’s smooth and “normal” functioning and needs to be intentionally addressed by leaders.

Second, we adopt a conceptual understanding of diversity that moves beyond the categorial framing of *otherness*. Following Wheeler (21, p. 60), we define diversity as “all the ways in which we differ and how those differences enable, enhance or inhibit the ability of individuals, groups and organizations to achieve individual, collective and/or organizational goals and objectives”. Building on this foundation, we understand diversity as the plurality of perspectives *and* diversity characteristics of groups and individuals (22).

Third, we approach diversity as an *anthropology of the otherwise* (23). In this sense we concentrate on the resource of translating a variety of *lifeworlds* (subjectively experienced everyday life of care workers) and perspectives on care work into an individual asset. This framing allows us to recognize and value different ways of *being* and *knowing*, rather that passively categorizing difference.

### Enhanced human capital framework

Rooted in economic analysis, human capital theory is centered around the “notion that an investment in human beings today has a payoff in the future” (24, p. 225). Through the lens of macroeconomics, human capital constitutes an asset, that is, a productive resource. Depending on the specificities of the production process, human capital might (partially) substitute but also complement the other characteristic resources – physical capital and land. Investment decisions treating the optimal accumulation of human capital over time are guided by microeconomic analyses, typically deploying methods of (opportunity) cost–benefit analysis. Within this dynamic framework individual characteristics and capabilities, education, skills, health, mobility, and experiences generate – productive – value at individual, organizational and societal levels. While classical human capital research focused on cognitive measures of human capital (25), modern human capital frameworks integrate non-cognitive skills or endowments (26). For example, personal(ity) traits, intrinsic motivation, diversity in perspectives or preferences are specified as non-cognitive dimensions of human capital (27). In the context of care work, multiplicity and heterogeneity of *lifeworlds* and heritage of healthcare workers and their manifold experiences systematically impact their perceptions of *good care* and also promote idiosyncratic knowledge-frameworks. In this regard, diversity can be embedded as a dynamic resource. A major reason for us to build on the human capital framework is that it integrates the conceptual frameworks and associated empirical approaches of skill formation, the roles of parental backgrounds and other socio-economic status variables and, in particular, the historically strong interconnection with the studies of social injustice and discrimination. Aiming at an interdisciplinary interface accounting for systemic and societal injustice we also adopt Bourdieu’s cultural capital theory. In this way we can approach diversity as human capital both as a matter of individual strategies *and* a matter of social structure and its reproduction.

### Care work of the future models

The transformation of care work is characterized by accelerating change across multiple dimensions. As Conner (28) argues, resilience emerges as the pivotal pattern enabling organizational change, particularly as the volume, momentum and complexity of that change continue to intensify. This acceleration necessitates leaders to develop new competencies for change management while simultaneously fostering environments that are adaptive and open to transformation. The future of care work requires fundamental transformation encompassing both quantitative expansion and qualitative evolution of the care workforce, while simultaneously addressing economic, ethical and demographic challenges (12). Healthcare projections for 2030 by major consulting companies like KPMG and Deloitte emphasise four key developments: (i) increased individual responsibility to manage their own health, (ii) personalised care approaches, (iii) value-enhancement programmes, and (iv) the integration of digital technologies, AI and the Internet of Medical Things alongside expanded health data utilisation. Although their predictions do not explicitly delineate the roles of care workers in this transformation, it is evident that care workers themselves are critical to facilitating changes beneficial to both patients and themselves, organizations, and the civic society. They are tasked with evolving both healthcare service delivery and their professional identities to meet future demands – a process that directly intersects with how diversity as an individual resource can be mobilized to navigate and shape these changes.

The intersection of these three fundamental concepts points to a necessary paradigm shift. While traditional DEI frameworks address historical injustices, they typically position diversity as a challenge requiring management rather than as an individual and organizational asset. By integrating diversity with human capital research, we can recognize how diverse perspectives, experiences, and *lifeworlds* constitute valuable resources at both individual and organizational levels. Given that care work of the future is prone to dramatic changes and demands a culture of diversity in terms of characteristics, skills and perspectives, a shift of perspective is essential: rather than managing diversity as an obstacle or additional challenge, this scoping review explores how it can be strategically deployed as a dynamic individual resource that enables healthcare workers to navigate transformation and shape future health care models.

The following figure presents the three main theoretical strands outlined above and their intersections. In this scoping review, we conceptualize diversity as an individual resource as a dynamic concept. Accounting for the interdisciplinary character of the review, this working model integrates diversity characteristics (drawing on traditional diversity-related research in healthcare), diversity in perspectives (drawing on a socio-anthropological perspective), and non-cognitive skills and endowments (drawing on an economic and human capital perspective). These three components jointly (in)form care workers’ lived experiences of diversity. At the centre of the model lies the process of translating static *differences* into dynamic *otherwise,* which in turn shapes the individual *lifeworlds*. Rather than being passively different, diversity as a resource simultaneously shapes and is shaped by the *lifeworld* – through the development of individual strategies and through social structures – feeding into the understanding and delivery of “*good care*” and enabling navigation through transformation toward future care models. This process goes hand in hand with the development of individual skills and capabilities as an investment that pays off over time (Figure 1).

**Figure 1:**
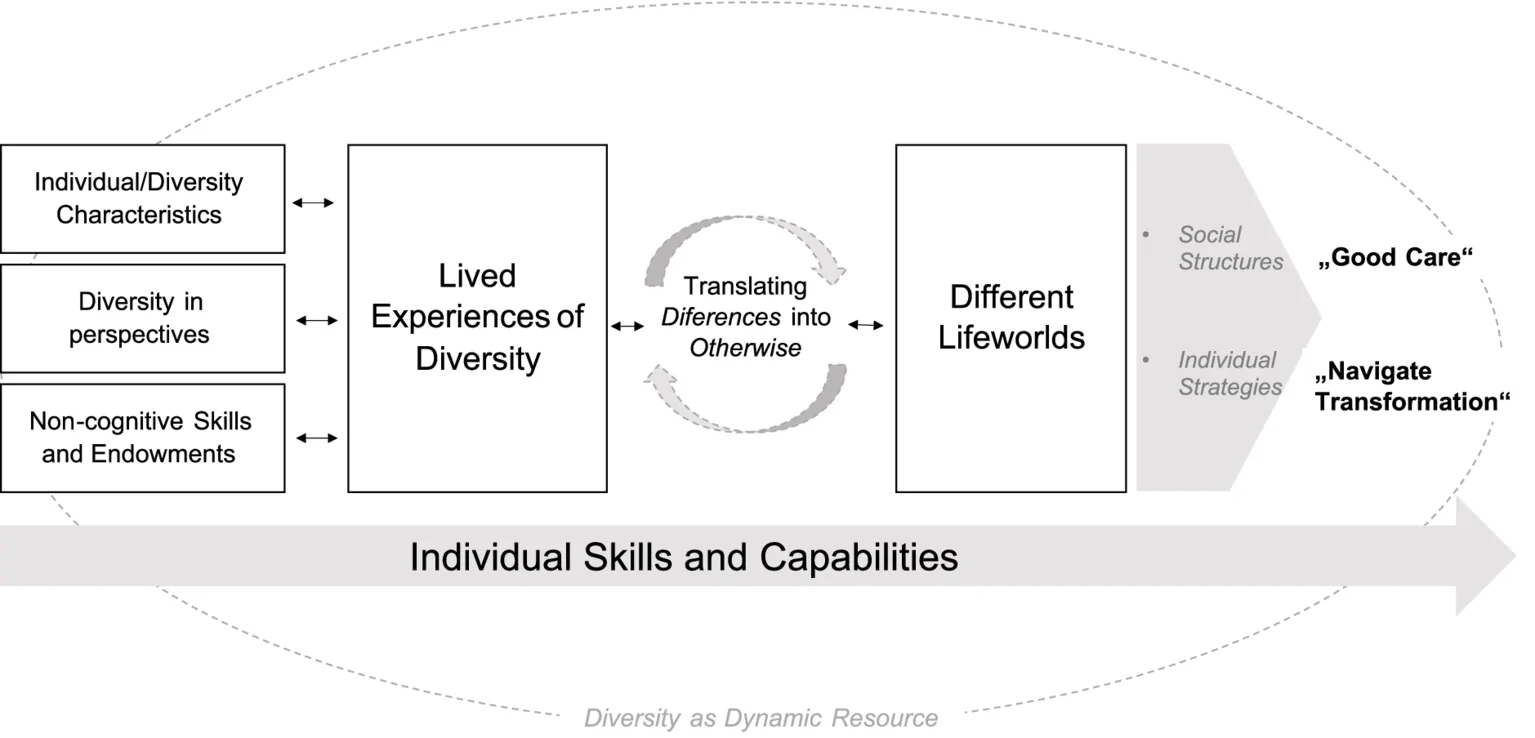
Conceptual model as starting point for operationalizing diversity as an individual resource in care.

As this scoping review maps evidence on how lived diversity experience is conceptualized and operationalized as an individual resource among healthcare workers, a preliminary search of MEDLINE, the Cochrane Database of Systematic Reviews and *JBI Evidence Synthesis* was conducted and no current or underway systematic reviews or scoping reviews on the topic were identified. In order to avoid positive bias, this review employs an interdisciplinary search and analysis strategy, which means that it reflects a broad variation of terms describing diversity-relevant aspects of care in the different disciplines. Furthermore, it aims at searching for studies that frame diversity both as an individual resource and/or as an obstacle, even though it concentrates not on the question of *if* but on the *how* diversity is deployed as an individual resource. This review also intends to synthesize findings and data on diversity of healthcare workers and to explore how these can be related to care work of the future. In doing so, it seeks to address a critical gap identified in the literature – namely, how healthcare workers and their employers can build a care and working culture that incorporates diversity. This review will provide foundational evidence to inform educational strategies, support workforce recruitment and shift institutional approaches from deficit-based frameworks toward asset-based models that specify diversity as crucial to achieving health equity and person-centered care.

## Methods

The proposed scoping review will be conducted according to the Joanna Briggs Institute (JBI) methodology (29) and the PRISMA Extension for Scoping Reviews (PRISMA-ScR) (30). Grey literature will be hand searched based on the search algorithm. The protocol is reported according to the Preferred Reporting Items for Systematic review and Meta-Analysis Protocols (PRISMA-P) (31). Moreover, it was registered with Open Science Framework (embargoed registration).

### Review question

What is known about diversity as an individual resource/obstacle among healthcare workers, and how do these resources align with competencies needed for the future of care work?

- How is lived diversity experience conceptualized as an individual resource/obstacle in care work?
- What types of diversity resources are identified, and how are they measured?
- How is diversity-as-resource treated within discussions of workforce transformation and the future of care work?
- What gaps exist in understanding diversity resources as future-relevant non-cognitive skills in care work?
- What chances exist in employing individual diversity resources in responding to the requirements of work of the future in the field of care?

### Inclusion criteria

#### Participants

The current review will consider studies that discuss diversity related to care workers, which includes nurses, social workers, home care workers, allied health professionals. Nursing students will be also included, provided they report on practical experience in care settings. The data on participants will be extracted in a diversity-sensitive manner, as far as the selected articles contain available data on participants age, gender, educational background and other diversity characteristics.

#### Conceptual framework

The concept that will be studied is lived diversity experiences as background characteristics, skills, knowledge, perspectives that care workers possess and deploy. This includes theories and empirical data on diversity as individual resource, whereby care workers actively use their diversity background as a resource, not just passively “being diverse.” This includes, e.g., personal diversity background having an impact on care delivery, multilingualism or -culturalism, migration background (as worker capability), LGBTQ+ identity informing care approaches, disability experiences informing care provision, or minority experience as knowledge base. Further dimensions that may emerge during data analysis will be also considered. The concept of diversity will be studied in an interdisciplinary approach. Thereby, theories on (care) culture, cultural and human capital and workforce transformation will be accounted for.

#### Context and setting

The current review will consider studies conducted in a health-care, educational or community-based setting. Studies conducted in controlled settings that suggest or evaluate interventions for improving diversity skills will be excluded, unless they focus on diversity of care workers. Studies that examine solely diversity of care receivers with no relation to care-workers diversity will be also excluded.

#### Types of sources

This scoping review will consider empirical and theoretical studies published in English or German since 2016 for inclusion. It will include both qualitative and quantitative studies that address at least one diversity factor among health care workers. This review will also consider descriptive observational study designs including case series or ethnographies for inclusion. In addition, systematic reviews that meet the inclusion criteria will also be considered. Text and opinion papers will not be considered for inclusion in this scoping review due to limitation in resources.

### Search strategy

The literature search strategy aims to locate published theoretical and empirical work on diversity as an individual resource among care workers. A four-step search strategy will be utilized in this review. First a preliminary limited search of MEDLINE (PubMed) was undertaken to identify articles on the topic and to refine the search strategy. We first tested a broad search term excluding the third (economic) theoretical dimension of the operationalized concept of diversity as an individual resource: (intersectionality OR diversity OR inclusivity OR inclusive OR intersectional OR identity) AND (nurse* OR “nursing student*” OR “care worker*” OR “healthcare worker*” OR “healthcare professional*” OR “medical student*” OR “allied health*” OR “social care*” OR “long-term care*”) AND (multicultural* OR multilingual* OR disabilit* OR Indigenous OR neurodivergent OR “lived experience*” OR “cultural background*” OR migrant* OR refugee* OR ethnic* OR racial OR race OR LGBTQ* OR gender* OR age* OR socioeconomic* OR religio*). This yielded approximately 14,200 results. After screening the first 200–400 (sorted by relevance), we identified a high proportion of irrelevant results and marked the most relevant studies. We than added the third theoretical block (economic/human capital dimension) to the search query and confirmed that none of the previously identified relevant studies was omitted from the results. This refined, three-block search string yielded 734 results and was adopted as the preliminary search strategy. Second, the text words contained in the titles and abstracts of relevant articles, and the index terms used to describe the articles were used to develop a full search strategy for MEDLINE/PubMed, PsycINFO, Scopus and Web of Science. Each query algorithm, including all keywords and index terms, was adapted for each database to account for differences in indexing and data-base specific functionality (provided in Supplementary Appendix I). The search was limited to studies published from 2016 onward, corresponding to the adoption of the WHO’s Global Strategy on Human Resources for Health: Workforce 2030 (32). This strategy marked a significant shift in global health workforce policy by addressing skill-mix imbalances, workforce maldistribution, and the need to strengthen the capacity and diversity of health and care workforce as a part of a broader agenda to achieve health equity. Studies published before this milestone were considered less likely to reflect contemporary policy and conceptual context in which diversity is discussed as a workforce-related resource. The search is further restricted to studies published in English and German, reflecting the language proficiency of the review team and ensuring accurate data extraction and synthesis. Third, grey literature will be included through hand search in the following sources: WHO (future health workforce), McKinsey Global Institute (future of work reports), World Economic Forum (Future of Jobs reports) and OECD (skills and workforce development). Finally, through citation chaining by reviewing reference lists of key papers, additional papers will be added to the body of found literature.

### Study selection

Following the search, all identified citations will be collated and uploaded into the citation management system Citavi 7.0. (2025) and duplicates removed. Following a pilot test, titles and abstracts will then be screened by two or more independent reviewers for assessment against the inclusion criteria for the proposed review. The inclusion and exclusion criteria that will be applied in the review are displayed in Table 1.

**Table 1:** Inclusion and exclusion criteria.

Inclusion criteria	Exclusion criteria
Studies included in the review must address diversity as individual resource/obstacle in care work AND/OR its relationship to: Future skills or competencies Workforce transformation Care models Technology integration Innovation in care delivery Adaptability and change management Empirical studies (quantitative, qualitative, mixed methods) Theoretical or conceptual papers Published since 2016 In English or German	Studies focusing only on organizational/representative diversity without individual-level analysis Studies about diversity training programs, status quo measurement of diversity competence or effectivity of DEI initiatives Patient diversity, culture-sensitive care delivery or health disparities research (unless worker diversity is examined) Pure discrimination/bias studies (unless they address diversity as resource) Opinion pieces without theoretical/empirical grounding, research protocols or preliminary reports

The screening procedure will follow a two-stage approach. In Stage 1, two independent reviewers (M.L. and V.C.) will screen titles and abstracts using Citavi 7.0., applying liberal inclusion approach where in case of uncertainty studies will be included. Exclusion reasons will be systematically tracked. In Stage 2, the reviewers will conduct a full text screening (also using Citavi 7.0) of all studies passing Stage 1, applying the complete eligibility criteria and documenting detailed reasons for exclusion. Throughout both stages, inter-rater agreement will be monitored to ensure consistency in decision making. Any disagreements that arise between the reviewers at each stage of the selection process will be resolved through discussion, or with an additional reviewer. While Citavi 7.0. will be used to manage references and support screening, coordination between reviewers will be conducted manually. The screening process will be documented in a PRISMA flow diagram and will be reported in full in the final scoping review.

### Data extraction

Data will be extracted from papers included in the scoping review by two independent reviewers using a data extraction tool developed by the reviewers (see Supplementary Appendix II). Data charting will be performed and stored using Microsoft Excel by the reviewers. The following information will be collected: (1) study characteristics including authors, publication year, country, study design and methodology, setting (e.g., hospital, community), and sample size and characteristics; (2) population details such as professional roles, demographics, and types of diversity represented; (3) conceptual information including how diversity is conceptualized, any theoretical frameworks employed, and specific diversity dimensions addressed (e.g., cultural, linguistic, LGBTQ+, disability); (4) operationalization and measurement approaches, including quantitative instruments, qualitative methods used to assess diversity-as-resource, and whether measures were self-reported or observed; (5) outcomes and findings, encompassing the types of outcomes examined (e.g., patient care quality, worker wellbeing, job satisfaction, cultural competence), key findings related to diversity as a resource, and connections to the Care Work of the Future framework; and (6) reviewer notes on study quality, discrepancies and other relevant information. As a scoping review does not require a study quality appraisal, in this case, notes will be used only descriptively to map rigor across the field and not as an inclusion/exclusion filter. The draft data extraction tool will be modified and revised as necessary during the process of data extracting. Any disagreements that arise between the reviewers will be resolved through discussion, or with an additional reviewer. If appropriate, authors of papers will be contacted to request missing or additional data, where required.

### Data analysis and presentation

The extracted data will be presented in a tabular form, accompanied by a narrative synthesis that describes how the results relate to the aim of this review. Additionally, thematic analysis will be used to better understand and categorize the data. The three steps thematic synthesis will follow Thomas and Harden’s approach (33), whereby (1) line-by-line coding will take place in a concept driven way independently by both authors; (2) the codes will be grouped into descriptive categories, which will be then (3) interpreted to answer the review questions. Steps 2 and 3 of the analytical process will be performed in a collaborative manner by both authors. Disagreements will be resolved by a discussion and discrepancies will be transparently protocolled throughout the whole process. We will use the PRISMA-ScR checklist to report the scoping review.

### Changes to the study protocol

Changes to the study protocol will only be made if needed and will be reported accordingly.

## Discussion

This scoping review is expected to shift the focus from organizational-level diversity research to research on how diversity contributes as an individual resource in care work contexts. The review will map how diversity-as-resource is theoretically understood and operationalized across disciplines, identifying existing measurement approaches and potentially revealing critical gaps in current assessment methods. By synthesizing evidence on how care workers’ lived diversity experiences are deployed as valuable competencies in care delivery, this review will provide foundational knowledge to inform workforce transformational strategies (e.g., skills development, technology integration, organizational design, culture and leadership, etc.). Expected outcomes include articulating evidence-based approaches for integrating diversity as an individual human capital/capability dimension into educational curricula, recruitment and retention initiatives and organizational polices.
