# Supplementary material for: Diversity as an individual resource in care work of the future: a scoping review protocol
Source: Front Public Health. 2026 Oct 20;14:1892688. doi: 10.3389/fpubh.2026.1892688 (PMC13728859; doi:10.3389/fpubh.2026.1892688)
Supplement: Supplementary file 1 [file Supplementary_file_1.docx]

# Appendices

### Appendix I: Search strategy

| *Database* | *Search Strategy* |
| --- | --- |
| MEDLINE/PubMed | Abstract: (intersectionality OR diversity OR inclusivity OR inclusive OR intersectional OR identity) AND (nurse* OR "nursing student*" OR "care worker*" OR "healthcare worker*" OR "healthcare professional*") AND (multicultural* OR multilingual* OR disabilit* OR Indigenous OR neurodivergent OR "lived experience*" OR "cultural background*") AND (competenc* OR resource* OR strength* OR capabilit*) |
| PsycINFO | TI(intersectionality OR diversity OR inclusivity OR inclusive OR intersectional OR identity) AND (nurse* OR "nursing student*" OR "care worker*" OR "healthcare worker*" OR "healthcare professional*") AND (multicultural* OR multilingual* OR disabilit* OR Indigenous OR neurodivergent OR "lived experience*" OR "cultural background*") AND (competenc* OR resource* OR strength* OR capabilit*)  Limits: English; Article; Last 10 years  Exclusion: data collection, biodiversity, learning, covid-19, teachers, pandemic, curricula, data analysis, flowers and plants, species diversity, indigenous species, trees, forests, plant species, ecosystems, climate change, teaching methods, food |
| Scopus | (intersectionality OR diversity OR inclusivity OR inclusive OR intersectional OR identity ) AND ( nurse* OR "nursing student*" OR "care worker*" OR "healthcare worker*" OR "healthcare professional*" ) AND ( multicultural* OR multilingual* OR disabilit* OR Indigenous OR neurodivergent OR "lived experience*" OR "cultural background*" ) AND ( competenc* OR resource* OR strength* OR capabilit*)  AND subject areas excluded: Environmental Science, Agricultural and Biological Science, Computer Science, Biochemistry, Genetics and Molecular Biology, Business, Management and Accounting, Phamacology, Toxicology and Pharmaceutics, Dentistry, Mathematics, Earth and Planetary Sciences, Energy, Immunology and Microbiology, Chemistry, Material Science and the next one  AND limit the document type to: Articles; Chapters  AND limit the publication year to: ≥ 2016  AND limit the language to: Englisch  TITLE-ABS-KEY((intersectionality OR diversity OR inclusivity OR inclusive OR intersectional OR identity) AND (nurse* OR "nursing student*" OR "care worker*" OR "healthcare worker*" OR "healthcare professional*") AND (multicultural* OR multilingual* OR disabilit* OR Indigenous OR neurodivergent OR "lived experience*" OR "cultural background*") AND (competenc* OR resource* OR strength* OR capabilit*)) AND ( LIMIT-TO ( SUBJAREA,"NURS" ) OR LIMIT-TO ( SUBJAREA,"MEDI" ) OR LIMIT-TO ( SUBJAREA,"SOCI" ) OR LIMIT-TO ( SUBJAREA,"HEAL" ) OR LIMIT-TO ( SUBJAREA,"PSYC" ) OR LIMIT-TO ( SUBJAREA,"ARTS" ) OR LIMIT-TO ( SUBJAREA,"ENGI" ) OR LIMIT-TO ( SUBJAREA,"MULT" ) OR LIMIT-TO ( SUBJAREA,"NEUR" ) OR LIMIT-TO ( SUBJAREA,"DECI" ) OR LIMIT-TO ( SUBJAREA,"VETE" ) OR LIMIT-TO ( SUBJAREA,"ECON" ) ) AND ( LIMIT-TO ( DOCTYPE,"ar" ) OR LIMIT-TO ( DOCTYPE,"ch" ) ) AND ( LIMIT-TO ( LANGUAGE,"English" ) )  AND PUBYEAR > 2015 AND PUBYEAR < 2027 |
| Web of Science | (intersectionality OR diversity OR inclusivity OR inclusive OR intersectional OR identity) AND (nurse* OR "nursing student*" OR "care worker*" OR "healthcare worker*" OR "healthcare professional*") AND (multicultural* OR multilingual* OR disabilit* OR Indigenous OR neurodivergent OR "lived experience*" OR "cultural background*") AND (competenc* OR resource* OR strength* OR capabilit*)  AND limit the publication year to: ≥ 2016  AND language excluded: Portuguese |

### Appendix II: Data extraction instrument

| **Data Extraction** | |
| --- | --- |
| Paper ID / Reference Code |  |
| Primary Reviewer |  |
| Secondary Reviewer |  |
| Date of Extraction |  |
| Version / Iteration |  |
|  |  |
| Authors |  |
| Publication Year |  |
| Country / Countries |  |
| Study Design |  |
| Methodology |  |
| Setting |  |
| Sample Size |  |
| Sample Characteristics |  |
|  |  |
| Professional Roles / Occupations |  |
| Demographics of Workforce |  |
| Dimensions of Diversity Represented |  |
|  |  |
| How Diversity is Conceptualized |  |
| Theoretical / Conceptual Framework |  |
| Specific Diversity Dimensions Addressed |  |
| Definition of Diversity Used (if stated) |  |
| Framing of Diversity as Resource |  |
|  |  |
| Quantitative Instruments Used |  |
| Qualitative Methods Used |  |
| Mixed-Methods Approach |  |
| Self-Reported vs. Observed Measures |  |
| How Diversity-as-Resource is Operationalized |  |
| Reliability / Validity Noted |  |
|  |  |
| Types of Outcomes Examined |  |
| Key Findings: Diversity as Resource |  |
| Direction of Effect (if applicable) |  |
| Moderating / Mediating Factors Noted |  |
| Connection to Care Work of the Future Framework |  |
| Limitations Noted by Authors |  |
| Gaps / Future Research Identified |  |
|  |  |
| Quality / Rigour Assessment |  |
| Discrepancies Between Reviewers |  |
| Data Requested from Authors? |  |
| Additional Notes |  |
